# Enrichment of Glial Cells From Human Post-mortem Tissue for Transcriptome and Proteome Analysis Using Immunopanning

**DOI:** 10.3389/fncel.2021.772011

**Published:** 2021-12-13

**Authors:** Anna Nolle, Irene van Dijken, Ciril M. Waelti, Daniela Calini, Julien Bryois, Emmanuelle Lezan, Sabrina Golling, Angelique Augustin, Lynette Foo, Jeroen J. M. Hoozemans

**Affiliations:** ^1^Department of Pathology, Amsterdam Neuroscience, Amsterdam UMC, Amsterdam, Netherlands; ^2^Roche Pharma Research and Early Development, Neuroscience and Rare Diseases Discovery and Translational Area, Roche Innovation Center Basel, Basel, Switzerland; ^3^Pharmaceutical Sciences, Biomarkers, Bioinformatics and Omics and Pathology (MT, JL, AA), Roche Innovation Center Basel, Basel, Switzerland

**Keywords:** immunopanning, human microglia, human oligodendrocytes, human astrocytes, post-mortem human brain, purification protocol

## Abstract

Glia cells have a crucial role in the central nervous system and are involved in the majority of neurological diseases. While glia isolation techniques are well established for rodent brain, only recent advances in isolating glial cells from human brain enabled analyses of human-specific glial-cell profiles. Immunopanning that is the prospective purification of cells using cell type-specific antibodies, has been successfully established for isolating glial cells from human fetal brain or from tissue obtained during brain surgeries. Here, we describe an immunopanning protocol to acutely isolate glial cells from post-mortem human brain tissue for e.g. transcriptome and proteome analyses. We enriched for microglia, oligodendrocytes and astrocytes from cortical gray matter tissue from three donors. For each enrichment, we assessed the presence of known glia-specific markers at the RNA and protein levels. In this study we show that immunopanning can be employed for acute isolation of glial cells from human post-mortem brain, which allows characterization of glial phenotypes depending on age, disease and brain regions.

## Graphical Abstract

Schematic overview of the protocol for human glial-cell enrichment.

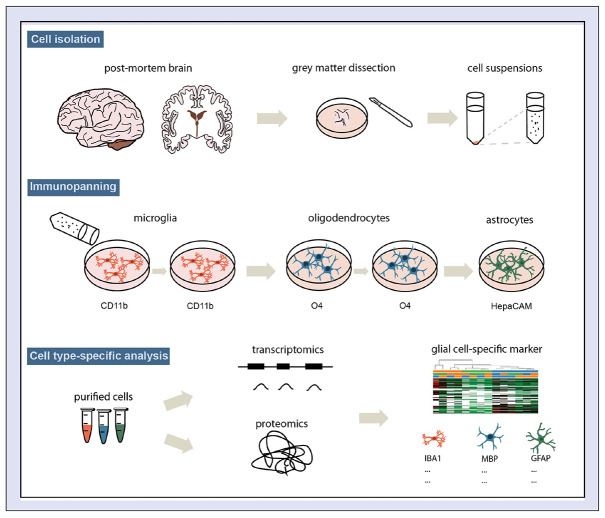

## Introduction

Glial cells, especially microglia, oligodendrocytes and astrocytes represent more than 50% of the brain cells and play a crucial role in tissue repair and diseases of the central nervous system. Brain tissue damage leads to acute activation of glia, which initiates protective processes to maintain the integrity of the neuronal network (Sofroniew and Vinters, 2010; Anderson et al., 2016). In disease, brain tissue displays a chronic activation profile of glia, which involves both protective and neurotoxic markers (Heneka et al., 2015; Zuchero and Barres, 2015). Glial cells therefore play a dual role in disease and the functions of activated glia may depend on the stage of the disease (Molofsky et al., 2012; Heneka et al., 2015; Pekny et al., 2016; Priller and Prinz, 2019). In addition, glial risk genes identified in GWAS on Alzheimer’s disease (AD) and Parkinson’s disease (PD) cases indicate that glial dysfunction is a driving factor in the development and progression of CNS diseases (Bohlen et al., 2019; Hammond et al., 2019; Jansen et al., 2019). Although glial cells represent a potential avenue for treatment, their exact function in brain diseases is still unclear, and more information is needed to understand when and how glial cells could be targeted for pharmacological intervention.

Microglia are the primary immune cells of the nervous system. They permanently scan the brain parenchyma for insults and react to changes in the microenvironment by adopting different activation states (Nimmerjahn et al., 2005; Tremblay et al., 2011; Li and Barres, 2018). These are traditionally characterized by their pro-inflammatory (M1) and anti-inflammatory (M2) cytokine profile, which are accompanied by morphological changes (Heneka et al., 2015). This classification is derived from *in vitro* situations and may insufficiently describe the broad spectrum of activation states in the tissue environment (Jurga et al., 2021). In addition, microglia interact with neurons early in development and regulate the neuronal network by synaptic pruning (Paolicelli et al., 2011; Schafer et al., 2012; Kettenmann et al., 2013). Astrocytes are responsible for a variety of homeostatic and metabolic processes in the brain. Their close connection to the neurons ensures neurotrophic support and regulates synaptic transmission and plasticity (Zuchero and Barres, 2015; Allen and Eroglu, 2017). Similar to the activation states attributed to microglia, different types of reactive astrocytes have been reported in neurodegeneration (A1) and injury (A2) (Liddelow et al., 2017). In a recent publication, the A1/A2 classification of reactive astrocytes was revised and the authors stated that the term “reactive astrocytes” describes multiple states that astrocytes can adopt in response to the tissue environment (Escartin et al., 2021). Oligodendrocytes produce the myelin sheath insulating the axon, which is fundamental for action potential propagation in the CNS. Recently, also trophic neuronal support has been added to oligodendrocytes’ function (Nave and Werner, 2014; Zuchero and Barres, 2015).

In summary, glial activation in disease is by far more complex than the traditional classification of activations states. In this regard, advances in transcriptomic and proteomic techniques have proven to be very valuable because they enable analyses of cell type-specific expression profiles. Over the last years, datasets accumulated mainly from studies using glia derived from mouse models since the source of human glial cells is limited. Recently, comparative studies revealed substantial differences in the transcriptome of rodent and human glial cells, which urges the use of human models in translational research (Zhang et al., 2016; Galatro et al., 2017; Gosselin et al., 2017; Zhou et al., 2020). Further, transcriptomic analyses of glial cells, that were isolated and cultured from post-mortem tissue showed that these cells underwent drastic changes *in vitro* (Gosselin et al., 2017). This might affect their disease-specific signature and therefore analysis of acutely isolated cells are advantageous for the assessment of their *in vivo* expression profile.

Recently, immunopanning has been established as a promising method for the prospective purification of human glial cells from fetal or surgically dissected tissue (Zhang et al., 2016). The protocol was adapted from a purification method for rodent astrocytes and involved passing the isolated cell suspension over petri dishes coated with antibodies specific for microglia, oligodendrocytes or astrocytes, respectively (Foo et al., 2011). Here, we show for the first time that glial cells can be successfully isolated from post-mortem human adult brain using an immunopanning-based protocol. Based on the isolation method published by Zhang et al. (2016) we established a protocol to selectively isolate cells from cortical gray matter and prepared samples in parallel for RNAseq and MassSpec. Our analysis confirmed the presence of glia-specific markers in the respective fraction. This method facilitates comparative studies of glial cells derived from healthy, aged and diseased post-mortem human adult brain. Ultimately, a combined transcriptomic and proteomic approach will potentially lead to the identification of new disease markers and contribute to understanding the role of glia in pathology.

## Materials and Methods

### Tissue Isolation

Cortical gray matter (inferior frontal gyrus 2 and 3) was acquired during autopsy according to the standard protocols of the Netherlands Brain Bank (NBB), Netherlands Institute for Neuroscience (NIN), Amsterdam. Post-mortem brain tissue was collected from donors with written informed consent for brain autopsy and the use of brain tissue and clinical information for research purposes (see Table 1 for donors). The brain donor program of the NBB was approved by the local medical ethics committee of the VU university medical center (Ref#2009/148).

**TABLE 1:** Donors used for cell isolation (AD Alzheimer’s disease, GM gray matter, HAT Huntington, PMD Post-mortem delay).

Cases	Age	M/F	PMD	Diagnosis	Tissue	Analysis
1	44	F	4:20	HAT	GM	qPCR
2	96	M	4:50	ctrl	GM	qPCR
3	89	F	6:00	ctrl	GM	qPCR
4	91	F	9:30	ctrl	GM	qPCR*
5	94	F	4:35	ctrl	GM	Sequencing/proteomics
6	102	F	3:55	ctrl	GM	Sequencing/proteomics
7	98	M	4:45	AD	GM	Sequencing/proteomics/pictures

*Whole brain (WB) tissue was not available for qPCR from case 3; WB tissue from case 4 was used.

### Isolation of Glial Cells From Gray Matter

At autopsy cortical brain tissue (5–15 grams) was collected in collection medium [Dulbecco’s modified Eagle medium (DMEM) and HAM F10 1:1 supplemented with 50 μg/ml Gentamycin, (Gibco)] and stored at 4°C for maximally 24 h. Glia cells from gray matter tissue were isolated according to the protocol published by Zhang et al. (2016). In short, Earle’s Balanced salt solution (EBSS, Sigma) supplemented with 0.45% D (+) Glucose (Gibco) and with 0.5 mM EDTA (Millipore) (enzyme solution) or without EDTA (inhibitor solution) were equilibrated with 5% CO_2_ at 37°C overnight. Papain (Worthington, 400U for 0.5 gram gray matter tissue) and 5.5 mM L-cysteine (Sigma) was added freshly to the enzyme solution (ES +) and incubated at 37°C for 15 min for enzyme activation. Cortical tissue collected in collection medium was washed in phosphate-buffered saline (PBS) and 1 gram of gray matter was dissected and homogenized into fine pieces with a scalpel. Subsequently, tissue was digested in ES + for 90 min at 5% CO_2_ and 37°C and swirled every 10 min. Tissue was transferred to a 15 ml tube and ES + was carefully removed after chunks had settled on the bottom of the tube. For stepwise inhibition of the enzymatic digestion, tissue was washed 3 times with inhibitor solution supplemented 0.1% BSA (Sigma) and 0.1% Ovomucoid (Worthington) (Low Ovo). Low Ovo supplemented with 56.5 units/ml DNase was added and tissue was triturated into a single cell solution. Next, cell suspension was carefully layered above the inhibitor solution supplemented with 0.7% BSA and 0.7% Ovomucoid (High Ovo). Full inhibition of papain activity was ensured by centrifuging the cell suspension at 300 × g for 5 min through High Ovo. The cell pellet was resuspended in PBS supplemented with 0.02% BSA and 12.5 units/ml DNase (Sigma) (panning solution), passed through a 70 μm filter and used for immunopanning. For side-by-side isolation for transcriptomics and proteomics amounts were doubled (Supplementary Table 1).

### Immunopanning

15 mm × 150 mm polystyrene Petri dishes (Sigma) were coated with secondary antibody diluted in 50 mM Tris-HCl pH 9.5 at 4°C overnight or at 37°C for 2 h (Table 2). After three washing steps with PBS, dishes were incubated with primary antibody (CD11b, O4, HepaCAM) in PBS supplemented with 0.2% BSA at room temperature for 1.5–2 h. To efficiently pan out microglia and oligodendrocytes from the cell suspension before incubating with HepaCAM, two sets of CD11b and O4 coated plates were prepared. For side-by-side isolation for transcriptomics and proteomics amounts were doubled. The cell suspension was sequentially passed over the CD11b plates, over the O4 plates and finally over the HepaCAM plate. The respective incubation steps were performed for 15 min on each plate at room temperature. After incubation with cell suspension plates were washed 8 times with PBS and cells were scraped and collected in lysis buffer consisting of 50 mM Tris-HCl pH 8.5, 8 M Urea and Protease inhibitor cocktail (Roche) for proteomics analysis or RTL buffer (Qiagen) supplemented with 1% β-Mercaptoethanol for RNA analysis.

**TABLE 2:** Antibodies used for immunopanning.

Antibody	Species	Source	Concentration
CD11b	Mouse	Biorad	1:750
CD45	Rat	BD Pharmingen	1:750
O4	Mouse	R&D systems	1:1,250
HepaCam	Mouse	R&D systems	1:750
Anti-mouse IgG + IgM (H + L)	Goat	Jackson ImmunoResearch	1:400
Anti-rat IgG (H + L) chain	Goat	Jackson ImmunoResearch	1:400

### qPCR Analysis

Cells were harvested after washing in panning buffer by scraping in QIAzol Lysis Rea gent (Qiagen) or collected by centrifugation after which the pellet was resuspended in QIAzol Lysis Reagent. RNA extraction was performed using the miRNeasy Mini Kit (Qiagen) according to the manufacture’s protocol. The NanoDrop 1,000 spectrophotometer (Thermo Scientific) was used to assess RNA concentration, purity and integrity. 0.1 μg of RNA in 15 μl volume was used for cDNA synthesis and cDNA was generated using SensiFAST™ cDNA Synthesis Kit (Bioline) according to the manufacture’s protocol. 1 μl cDNA of each sample was pipetted in triplicate into a 384-wells plate for UPL-based (UPL library, Roche) real-time qPCR using Light Cycler 480 system. Primers and UPL probe combinations are provided in Table 3 and SensiFAST Probe No-ROX kit (Bioline) was used for qPCR reaction (5 μl per well) according to the manufacture’s protocol. Advanced Relative Quantification analysis of the LightCycler 480 software was used for analysis.

**TABLE 3:** Primers (Eurogentec) and Probes (Universal Probe Library for Human probes, Roche) used for qPCR analysis.

Target gene	Sequence 5′–3′	Universal probe	Bp product length
ALDH1L1	Fw cagaccttccgctactttgc RV ggtctggcctggttgatg	22	74
CD68	Fw ggctggctgtgcttttct RV tttttgtgaggacagtcattcc	45	79
GAPDH	Fw tccactggcgtcttcacc RV ggcagagatgatgaccctttt	45	78
GFAP	Fw atcaactcaccgccaacag RV agcctcaggttggtttcatc	19	106
IBA1	Fw ttaatggaaatggcgatattga RV ttctttagctctaggtgagtcttgg	67	88
MBP	Fw agccctctgccctctcat RV cgggtggtgtgagtcctt	71	69

### RNA Sequencing

RNA was extracted using the RNAeasy Micro kit (Qiagen). Total RNA quality was assessed by TapeStation-4200 (Agilent Technologies, Inc.), and the RNA integrity number was taken into consideration for optimization of the library construction protocol. SMART-Seq Stranded kit (Takara Bio USA, Inc.) was used according to manufacturer’s instructions to prepare cDNA libraries starting by 10 ng of total RNA. The quality of the libraries was assessed by TapeStation-4200 and they were sequenced using a SP flowcell on the Illumina Novaseq 6000 sequencer to obtain 50 bp pair-end reads, with a sequencing depth of 50 million reads per sample.

Raw RNA-seq reads were processed with the ARMOR pipeline (PMID: 31088905) using the GRCh38 reference genome and gencode v34 annotation. Transcripts per millions for each gene were obtained using Salmon v1.2.0 (PMID: 28263959).

### Proteome Analysis

After thawing, samples were sonicated in a Bioruptor Instrument (Diagenode) for 10 cycles of 30 s and centrifuged at maximum speed (14,000 rpm, Eppendorf Centrifuge 5417R) to pellet cell debris. Supernatants were reduced with 5 mM DTT at 56°C for 30 min and alkylated in 50 mM iodoacetamide for 1 h. at room temperature. After a fourfold dilution in TEAB 50 mM pH8.8, samples were digested with trypsin at 37°C for 4 h. Resulting peptide samples were desalted on Sep-Pak^®^ Vac C18 Cartridges (Waters) according to the manufacturer’s instructions. Mass spectrometry analysis was performed on an Orbitrap Fusion™ Tribrid™ instrument coupled to an Easy nLC 1200™ (Thermo Fischer Scientific). Samples were injected on a 2 cm × 75 um Trap Column, in line with a 50 cm × 100 um PepMap 2 um column (ES803; Thermo Fischer Scientific) and separated by a 120 min gradient from 5–80% acetonitrile. Survey scans of precursors ions were acquired over an *m*/*z* range of 300–1,500, 120K resolution, AGC target of 2e5, maximum injection time of 100 msec. MS^2^ scans were acquired in top speed mode with 3 s cycles, on MS1 ions of charge state 2–6 using HCD activation with a collision energy of 30% and isolation window of 1.6 m/z. Data have been processed using Proteome Discoverer 2.4 (Thermo Fischer Scientific), searching on human Swissprot database (January 2020; 20,386 entries) in Mascot version 2.6. The following search parameters were used: (1) Trypsin P as protease with a maximum of two missed cleavages, (2) carbamidomethylation of Cys (+ 57.0214 Da) as fixed, and (3) oxidation of Met (+ 15.9949 Da) as variable modification. MS and MS/MS tolerances were set to 10 ppm and 0.5 Da, respectively.

## Results

To test whether we can selectively isolate glial cells from cortical post-mortem tissue, gray matter tissue from the inferior frontal gyrus dissected during autopsy was enzymatically digested with papain to generate a single cell suspension. Next, we pulled out selected glial cells using immunopanning, which involves a series of incubation steps of the single cell suspension on Petri dishes pre-coated with antibodies. Therefore, we adapted the protocol published by Zhang et al. (2016) and used an anti-CD11b antibody for microglia, an anti-O4 antibody for oligodendrocytes and an anti-HepaCAM antibody for astrocytes for sequential purification (see Graphical Abstract). We expect 2–4 million cells in total isolated from 1 gram of gray matter using a papain based protocol. By extrapolating the cell numbers counted using microscopy pictures of the panning dishes (Supplementary Figure 1), we estimated that panning with CD11b antibody and HepaCAM antibody resulted in cell numbers of at least 500,000 and 50,000 cells, respectively, panning with O4 antibody yielded at least 10,000 cells.

**FIGURE 1:**
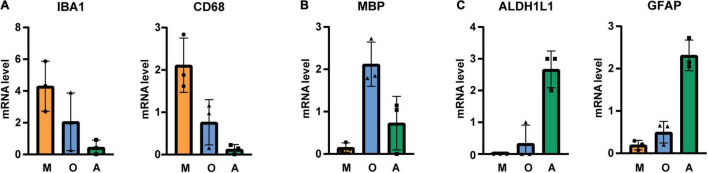
qPCR analysis of immunopanned cell samples for **(A)** microglia-, **(B)** oligodendrocyte-, and **(C)** astrocyte-specific transcripts. mRNA levels were normalized to GAPDH and values were averaged across different cell types and cell-specific signals were normalized to average. M microglia/macrophage sample, O oligodendrocytes sample, A astrocytes sample, *N* = 3 donors, shown is the mean ± SD.

Immunopanned cells were collected for qPCR analysis and samples from three donors (Table 1) were assessed for microglia (IBA1, CD68)-, oligodendrocyte (MBP)- and astrocyte (GFAP, ALDH1L1)-specific markers. Our analysis showed that microglia, oligodendrocytes and astrocytes markers were enriched in the respective sample (Figure 1). In comparison to whole gray matter lysates, microglial markers are highly increased while astrocytes and oligodendrocyte markers are only slightly higher (Supplementary Figure 2).

**FIGURE 2:**
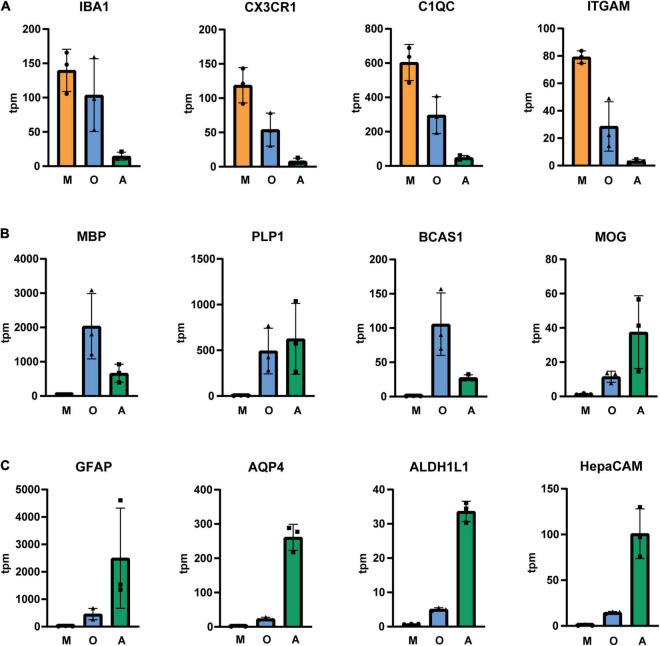
Tpm (transcripts per kilobase million) of glia-specific transcripts in immunopanned cell samples. Microglia/macrophage marker **(A)**, oligodendrocyte marker **(B)** and astrocyte marker **(C)**. M, microglia sample; O, oligodendrocyte sample; A, astrocyte sample, *N* = 3 donors, shown is the mean ± SD.

**FIGURE 3:**
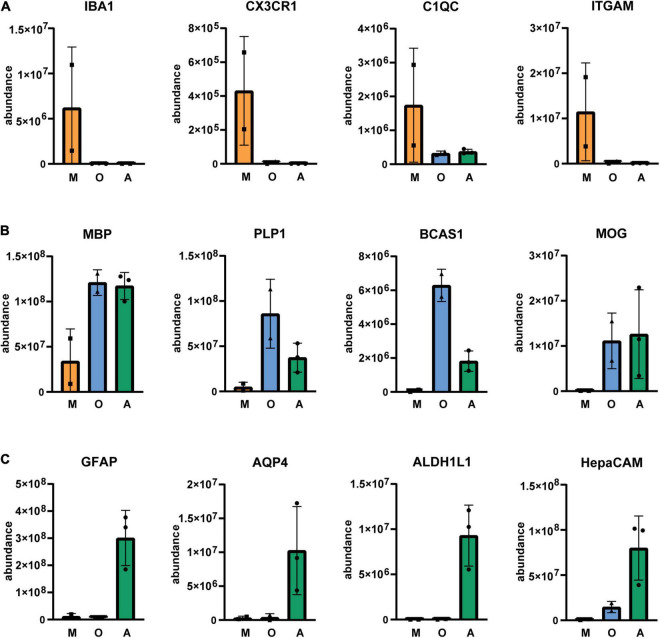
Relative abundance of glia-specific proteins in immunopanned cell samples. Microglia/macrophage marker **(A)**, oligodendrocyte marker **(B)** and astrocyte marker **(C)**. M, microglia sample; O, oligodendrocyte sample; A astrocyte sample. *N* = 3 for astrocytes, *N* = 2 for microglia and oligodendrocytes shown is the mean ± SD.

**FIGURE 4:**
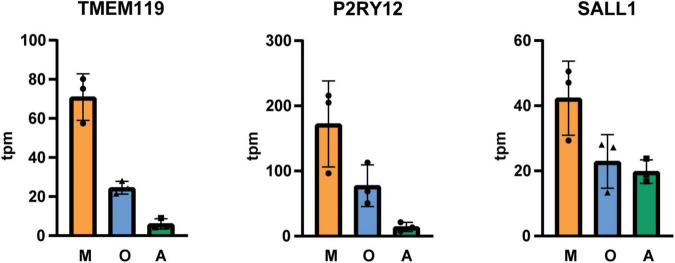
Tpm (transcripts per kilobase million) of microglia-specific transcripts in immunopanned cell samples. M microglia/macrophage sample; O, oligodendrocyte sample; A, astrocyte sample, *N* = 3 isolations, shown is the mean ± SD.

Next, we isolated cells by immunopanning and lysed side-by-side either in RLT lysis buffer (Qiagen RNA extraction kit) for RNA or UREA buffer for protein analysis. We collected samples from three isolations (Table 1) and analyzed them by RNA sequencing and MassSpec. We found that on both RNA and protein level, the known cell type-specific markers were enriched for microglia (IBA1, CX3CR1, C1QC, ITGAM), oligodendrocytes (MBP, PLP1, BCAS1, MOG) and astrocytes (GFAP, AQP4, ALDH1L1, HepaCAM), respectively (Figures 2, 3). Detection of microglia-specific marker that are not expressed in peripheral macrophages confirmed that we isolated microglia using the CD11b antibody (TMEM119, P2RY12, SALL1, Figure 4). In summary, the analysis showed that we were able to enrich microglia, oligodendrocytes and astrocytes from post-mortem gray matter tissue by our immunopanning protocol.

## Discussion

Analysis of brain tissue is crucial to understand cellular and molecular processes in neurological diseases. Whole brain tissue analysis might mask changes occurring in a specific cell type and therefore the use of isolated glia cells facilitates identification of cell-specific changes in disease. In the last years, emerging number of studies have been published using nuclear single cell sequencing isolated from post-mortem human brain in order to identify differentially expressed genes in disease (Galatro et al., 2017; Habib et al., 2017; Del-Aguila et al., 2019; Mathys et al., 2019; Zhou et al., 2020). In these analyses nuclei from whole brain tissue are isolated and cell types are identified based on the nuclear presence of cell type-specific transcripts. A recent publication showed that analysis of transcripts regarding the activation state of microglia are missed due to low nuclear abundances, emphasizing the need for alternative methods to complement these studies (Thrupp et al., 2020). Additionally, the quality of the RNA obtained from frozen tissue is not as high as from cells isolated from fresh tissue. Furthermore, while single nuclei sequencing has been incredibly informative, a large amount of mRNA is located in the cell bodies as well, and immunopanning allows for the isolation of the whole cell body.

Immunopanning is a selective isolation method that has been established to purify glia cells from rodent and human tissue (Foo et al., 2011; Zhang et al., 2016). Here, we applied immunopanning on post-mortem human gray matter tissue to isolate glia cells for transcriptome and proteome analysis.

We showed that we successfully isolated microglia and astrocytes with our immunopanning protocol. Although microglial numbers are sparse and make up about 5% of the cell population in the gray matter region (Mittelbronn et al., 2001), they can be highly enriched by immunopanning (Supplementary Figure 2). Mizee et al. (2017) reported an isolation method where they obtain 145,000 cells per gram gray matter from occipital cortex using CD11b coated magnetic beads. Here, we used papain digestion and omitted the percoll gradient centrifugation and obtained at least 500,000 cells per gram gray matter from frontal cortex on CD11b coated dishes. Our data confirm the presence of disease-associated microglial markers (e.g., APOE, CD74, CST3) which were detected with single cell sequencing but not with nuclear sequencing in the study by Thrupp et al. (2020). Further, the results of the transcriptome analysis indicate that we did not reach a complete depletion of microglia before incubation with O4 and HepaCAM coated plates (Figures 2, 4) and more optimization is needed to reach higher purity levels. Astrocytes are abundant in the gray matter but the yield of isolated astrocytes is lower in comparison to microglia and this might be due to lower viability of astrocytes in the post-mortem tissue (Sofroniew and Vinters, 2010). While astrocyte cultures from post-mortem brain could be established by a few proliferating astrocytes that form colonies and grow confluent within 2–4 weeks in culture (De Groot et al., 1997, 2001), isolation of post-mitotic mature astrocytes had been challenging in the past. More recently, with the discovery of new astrocyte surface markers, immunopanning and cell sorting protocols were used to isolate astrocytes from human tissue obtained from brain surgeries, but it was still an open question whether these methods can be used for isolation of astrocytes from human post-mortem brain tissue (Sun et al., 2013; Zhang et al., 2016). Here we showed that we were able to immunopan sufficient astrocytes from post-mortem tissue using an antibody against HepaCAM to perform RNA sequencing and proteome analyses.

Our data showed that oligodendrocytes were enriched in the respective sample in comparison to the microglia enriched sample. Oligodendrocytes were also detected in the astrocyte enriched samples, based on markers including surface antigen Claudin-11, which was only observed in the RNA dataset (Supplementary Figure 3). Previously Zhang et al. (2016) showed that their protocol depleted oligodendrocytes from the cell suspension prior to astrocyte panning (Zhang et al., 2016), which was required because HepaCAM is expressed by oligodendrocytes to a moderate extent. In the current study cell numbers were low after enrichment with the O4 antibody and qPCR results showed that enrichment of oligodendrocytes in the oligodendrocyte enriched sample is low in comparison to whole brain tissue (Supplementary Figure 2). We suspect that an inefficient depletion of oligodendrocytes by the O4 antibody, which targets Claudin-11, explains the low yield in the oligodendrocyte enrichment and presence of oligodendrocyte markers in the astrocyte enrichment. We expect overall low numbers of oligodendrocytes in the cell suspension due to their low abundance in the gray matter. For future studies, a screen for new oligodendrocyte surface marker may lead to identification of new antibodies that can be used for oligodendrocyte depletion.

Although immunopanning provides a fast and selective isolation of brain-derived cells from human post-mortem tissue, there are also limitations that apply to this technique. In addition to the expression analyses, we also tested whether we could culture microglia and astrocytes for functional assays after immunopanning these cells from gray matter. We were not able to establish a microglial or astrocyte culture after trypsinization of the immunopanned cells (Zhang et al., 2016) followed by earlier-established culture conditions for human primary glial cells (De Groot et al., 2001; Supplementary Table 1). It is conceivable that cells derived from post-mortem tissue are more vulnerable in comparison to cells isolated from tissue obtained during surgeries. It would be informative to address this in future experiments to compare tissue from both sources in this experimental setting. Performing the isolation directly after autopsy may increase viability but we were not able to shorten post-mortem delay or include tissue from surgeries due to practicalities at our department. Further, we used cortical gray matter tissue for immunopanning in this study while we and others showed in earlier studies that microglial cultures from post-mortem tissue are primarily established from white matter. Mizee et al. (2017) isolated microglia from both gray and white matter using a different isolation method and for both sources they confirmed viability of acutely isolated cells by FACS analyses. In line with other studies, viability was independent of donor age and post-mortem delay (Galatro et al., 2017; Mizee et al., 2017), however, viable cultures could only be established from white matter microglia. This indicates that the use of gray matter from post-mortem tissue for *in vitro* cultures is limited. So far, we have used the immunopanning protocol for subcortical white matter only in a pilot study, where the inclusion of a myelin removal step was necessary for successful immunopanning. Whether viable cultures from white matter or other brain regions could be established using immunopanning, remains to be investigated. In conclusion, the present immunopanning protocol cannot be applied to establish *in vitro* cultures from post-mortem gray matter.

Another limitation of this study could be the potential effect of the post-mortem delay and tissue treatment on gene expression and protein levels. Since this is almost unavoidable when working with human tissue, identified markers for glial cells or diseases identified using post-mortem human brain tissue must eventually be validated using *in vivo* and *in vitro* models. In addition, in this study we successfully obtained sequencing and proteomics data from three donors. Keeping in mind the general high variation in human donor material it is not possible to perform comparative analyses between controls and AD. Also, the amount of sample obtained using immunopanning could be a limitation. Here, we prepared cell lysates from one immmunopanning plate for RNAseq or Mass Spec analysis. However, for additional validation by Western blot, qPCR or similar methods, more plates could be prepared to reach sufficient amount of RNA or protein for analysis.

In conclusion, we have successfully established an immunopanning protocol which can be used for microglia, oligodendrocyte and astrocyte enrichment for transcriptome and proteome analysis from human post-mortem brain. This method facilitates studies of glial cell-specific profiles depending on age, disease and brain regions. Combined comparative analyses of the transcriptome and proteome will lead to the identification of glia cell-specific markers that can aid in the identification of new potential targets for therapy and diagnosis in neurodegenerative diseases.

## Data Availability Statement

The data presented in the study are deposited in the figshare repository, accession number 10.6084/m9.figshare.17032157 and 10.6084/m9.figshare.17032202.

## Ethics Statement

Ethical review and approval was not required for the study on human participants in accordance with the local legislation and institutional requirements. The patients/participants provided their written informed consent to participate in this study.

## Author Contributions

AN, LF, and JH designed the study. AN and ID performed glial cell purification. LF, CW, DC, JB, EL, SG, and AA performed the proteomics and RNA sequencing analysis. AN designed the figures and wrote the manuscript with input from all authors. All authors were involved in the interpretation of the analysis and approved the final version of the manuscript.

## Conflict of Interest

All Roche contributors are full time employees of F. Hoffman-La Roche, Basel, Switzerland. The authors declare that this study received funding from F.Hoffman-La Roche. The funder had the following involvement with the study: study design, data collection, data analyses, and writing of the article.

## Publisher’s Note

All claims expressed in this article are solely those of the authors and do not necessarily represent those of their affiliated organizations, or those of the publisher, the editors and the reviewers. Any product that may be evaluated in this article, or claim that may be made by its manufacturer, is not guaranteed or endorsed by the publisher.
