# Supplementary material for: Enrichment of Glial Cells From Human Post-mortem Tissue for Transcriptome and Proteome Analysis Using Immunopanning
Source: Front Cell Neurosci. 2021 Dec 13;15:772011. doi: 10.3389/fncel.2021.772011 (PMC8711556; doi:10.3389/fncel.2021.772011)
Supplement: Supplementary file 1 [file Data_Sheet_1.docx]

Supplementary Material


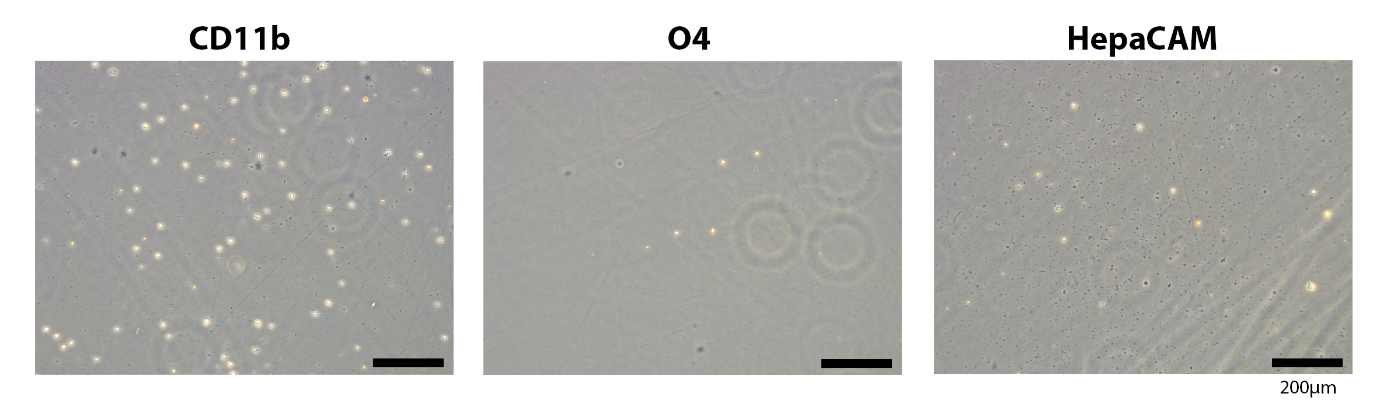


**Supplemental figure 1.** Representative pictures of cells bound on anti-CD11b, -O4 or -HepaCAM coated Petri dish plate after 15 min incubation with cell suspension.

**
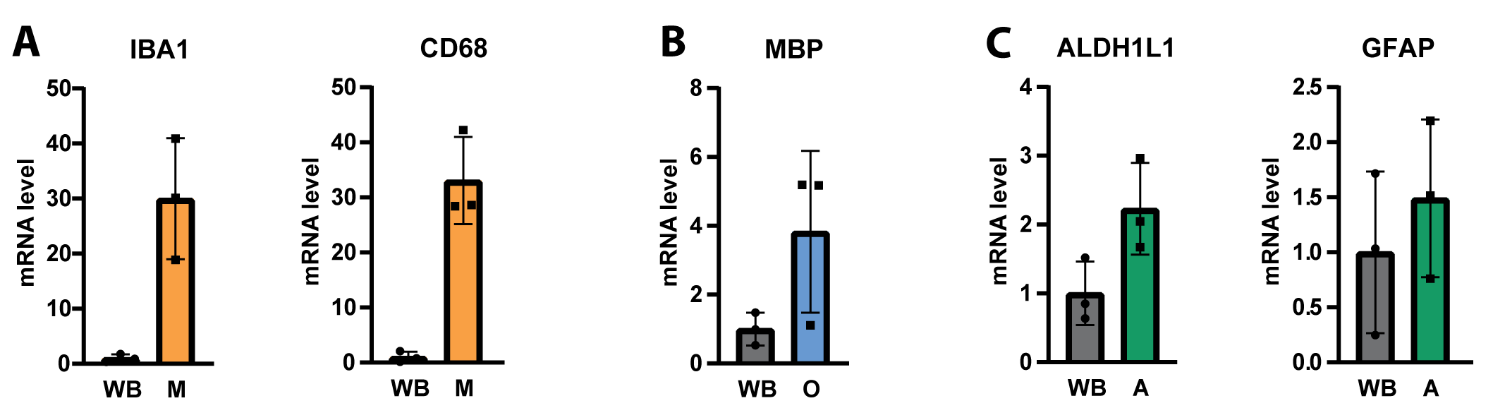
**

**Supplemental figure 2**. qPCR analysis of immunopanned cell samples for microglia (A), oligodendrocyte (B) and astrocyte (C) specific transcripts. mRNA levels were normalized to GAPDH and values were normalised to average of the whole brain (WB) sample (samples were not paired). M, microglia/macrophage sample; O, oligodendrocytes sample; A, astrocytes sample, N=3 isolations (shown is the mean +/- SD).


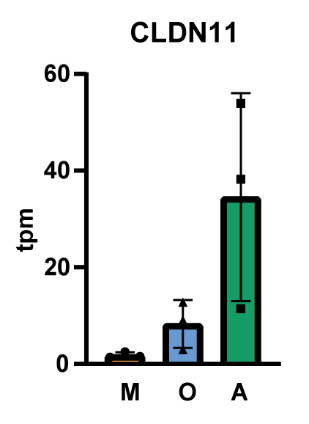


**Supplemental figure 3.** tpm (transcripts per kilobase million) of Claudin-11 (CLDN11) in immunopanned cell samples. M, microglia/macrophage sample; O, oligodendrocyte sample; A, astrocyte sample, N=3 isolations (shown is the mean +/- SD)
